# Pneumococcal Infective Endocarditis in two vaccinated Irish children; The Challenge of Non-Vaccine Serotypes and the Role of Host Immunity

**DOI:** 10.1007/s11845-026-04314-1

**Published:** 2026-04-09

**Authors:** Bailey Crowley, Sarah Geoghegan, Patrick Gavin, Cilian Ó Maoldomhnaigh, Bryony Treston, Robert Cunney, Aisling M. Flinn, Timothy Ronan Leahy, Mary Corcoran, Bridget Freyne

**Affiliations:** 1 Department of Paediatric Infectious Diseases & Immunology, Children’s Health Ireland, Crumlin and Temple Street, Dublin, Ireland; 2 Irish Meningitis & Sepsis Reference Laboratory, Children’s Health Ireland, Temple Street Hospital, Dublin, Ireland; 3 School of Medicine, University College Dublin, Dublin, Ireland

**Keywords:** Invasive Pneumococcal Disease, Pneumococcal Infective Endocarditis, Streptococcus Pneumoniae, Paediatric Infective Endocarditis, Drug Rash with Eosinophilia and Systemic Symptoms

## Abstract

**Introduction:**

Paediatric infective endocarditis is a rare but life-threatening condition. We describe cases of previously well, fully-vaccinated children who developed pneumococcal endocarditis.

**Cases:**

Case one, 11-year-old-boy, developed multi-focal osteomyelitis and septic arthritis, followed by infective endocarditisrequiring signifi cant surgical intervention of an aortic root abscess, coupled with marked ischemic changes onelectrocardiogram. These events identifi ed two unique, non-vaccine serotype Streptococcus pneumoniae isolates –23B and 20, respectively. Indicating two unique infections despite an extensive immunological workup, which wasunable to identify any inborn error of immunity. Case two, a three-year-old-girl, developed ocular manifestations ofinfective endocarditis with subsequent need for mitral valvuloplasty, with another non-vaccine serotype,Streptococcus pneumoniaeisolate – serotype 31, identifi ed as the off ending organism. Additionally, her treatmentcourse was complicated by a diagnosis of Drug Rash with Eosinophilia and Systemic Symptoms. In this casehowever, we identifi ed a defect in the alternative complement pathway activity.

**Conclusions:**

These cases highlight the increasing burden of invasive pneumococcal disease caused by non-vaccine serotypes. Wealso detail Irish national data which demonstrates a rise in non-vaccine serotype invasive pneumococcal disease inchildren since 2008. This report highlights the importance of expanded vaccine programs and the role of hostimmunity in invasive disease.

**Supplementary Information:**

The online version contains supplementary material available at 10.1007/s11845-026-04314-1.

## Introduction

Paediatric infective endocarditis (IE) is a rare but life-threatening condition. The most common organisms include viridians group streptococci (20–43%), *Staphylococcus aureus* (27–57%) and coagulase-negative staphylococci (2–14%). *Streptococcus pneumoniae* accounts for only 3–7% of cases [1]. Here, we present two cases of pneumococcal IE in children, both of whom had received pneumococcal vaccination in line with their respective national schedule and who had no history of underlying cardiac disease [2]. We present the clinical features of this rare but devastating paediatric infection and a discussion on the role of non-vaccine serotypes and host immunity.

## Case 1

An 11-year-old-boy presented with fever > 38 °C and a one-week history of multifocal joint pain involving the left knee, shoulder and neck. MRI confirmed multifocal osteomyelitis and septic arthritis. He had signs of septic shock and was admitted to the Paediatric Intensive Care Unit (PICU). He was commenced empirically on cefotaxime, clindamycin and vancomycin. The peripheral blood culture became positive at 16 h for *Streptococcus pneumoniae.* The patient had a normal echocardiogram and repeat blood culture on day 2 was sterile. He recovered well and discharged from PICU within 48 h. On day 5 he was discharged to outpatient parenteral antimicrobial therapy with ceftriaxone with a plan to complete 2 weeks prior to consideration for an oral switch. He developed delayed type hypersensitivity to ceftriaxone on day 12 of treatment and was switched to oral clindamycin 10 mg/kg tds to complete 6 weeks of therapy. Following discontinuation of therapy he developed left knee pain. Repeat MRI was consistent with osteochondritis dissecans and arthroscopy with sampling for histopathology was not consistent with osteomyelitis and the associated tissue culture was sterile.

One month later he presented with high fever, diarrhoea and generalised myalgia. He had a loud pansystolic murmur on auscultation and his peripheral blood culture was again positive for *streptococcus pneumoniae*. Echocardiogram confirmed aortic regurgitation, depressed left ventricular function, and a 1.7 × 2.4 cm aortic root abscess. Within the next 24 h he developed severe chest pain and marked ischemic changes on electrocardiogram. He had emergency surgery to drain the aortic root abscess and ultimately reconstruct the aortic root and valve. He was treated as an inpatient with course of IV cefotaxime before another delayed hypersensitivity reaction forced a switch to ertapenem. He completed 6 weeks total IV therapy. Post discharge, it was confirmed that the two blood culture isolates of *streptococcus pneumoniae* were different non-vaccine serotypes (23B and 20, respectively) and had different penicillin MICs. Serotype 23B demonstrated an MIC of 0.5 mg/L and, thus considered intermediate sensitivity/non-meningitis or resistant/meningitis according to EUCAST, while serotype 20 was susceptible with an MIC of 0.03 mg/L. An extensive immunological work-up including detailed functional immunological testing and next-generation sequencing of genes associated with immunodeficiency was undertaken and revealed no specific defect. The patient remains well on prophylactic amoxicillin.

### Case 2

A 3-year-old-girl presented to a Paediatric ED while on holidays from England with a one-week history of fever and a three-day history of a swollen erythematous right eye. She was previously healthy and fully vaccinated. She was empirically commenced on ceftriaxone and flucloxacillin and her peripheral blood culture was positive for *streptococcus pneumoniae.* Ophthalmological investigation noted debris, exudate, retinal detachment and Roth spots. Echocardiogram suggested mitral valve endocarditis and she was transferred to our centre for Cardiology and Cardiothoracic input. Her repeat echocardiogram confirmed a large vegetation on the mitral valve with perforation leading to severe mitral regurgitation, a severely dilated left atrium, a mild dilation of the right atrium, further moderate dilation of the left ventricle, and pulmonary and tricuspid valve regurgitation. She underwent vegetation removal and valvuloplasty. Three weeks into treatment with IV ceftriaxone she developed fever, rigors, a diffuse maculopapular rash associated with an abnormal inflammatory markers and hypereosinophilia. Ultimately a diagnosis of drug rash with eosinophilia and systemic symptoms was made, with treatment changed to IV amoxicillin and vancomycin. In this case the *streptococcus pneumoniae* serotype 31 was confirmed, another non-vaccine serotype. Alternate complement pathway activity was absent, suggesting a defect in this pathway.

## Discussion

Paediatric IE is estimated to occur in 0.43–0.69/100,000 children annually [3]. Risk factors are typically divided into congenital and acquired. Congenital heart disease is the pre-disposing factor in up to 64% of cases in in a 20-year-single-center observational study [4], whereas the detailed impact of acquired risk factors including indwelling catheters, and both chemotherapy-induced and primary immunodeficient states is less clear [3, 4].

While extracardiac manifestations of IE are rare in children, it is recognised that *Streptococcus pneumoniae* tends to lead to more fulminant disease as seen in these cases [1]. The American Heart Association recommends; a 4-week course of penicillin G or ampicillin for sensitive isolates but notes that in adults, a 4-week course of ceftriaxone is recommended. It is advised that infections by isolates with penicillin MIC > 0.5 should be managed in collaboration with infectious disease specialists. In cases where β-lactams are not tolerated, vancomycin and gentamicin are considered [1].

The life-threatening infections described here were caused by non-vaccine serotypes in children who had received the 13-valent pneumococcal conjugate vaccine (PCV13) as per the local schedules. Pneumococcal conjugate vaccines were introduced into the Irish and UK immunisation schedules in 2008 and 2006 respectively [2]. In both countries PCV7 (serotypes 4, 6B, 9 V, 14, 18 C, 19 F, and 23 F) was introduced first. Later in 2010 PCV7 was replaced with PCV13 in both countries (1, 3, 4, 5, 6 A, 6B, 7 F, 9 V, 14, 18 C, 19 A, and 23 F) [2]. Serotyping, molecular sequence-based typing, and reference susceptibility testing of invasive pneumococcal isolates is performed in the Irish Meningitis and Sepsis Reference Laboratory (IMSRL), based at CHI Temple Street, which also monitors changes in invasive pneumococcal disease (IPD) epidemiology to inform national immunisation policy. The isolate is tested using PCR and slide agglutination using antisera [5]. As previously mentioned, our two patients suffered three severe pneumococcal infections caused by non-PCV13 vaccine serotypes.

**Fig. 1:**
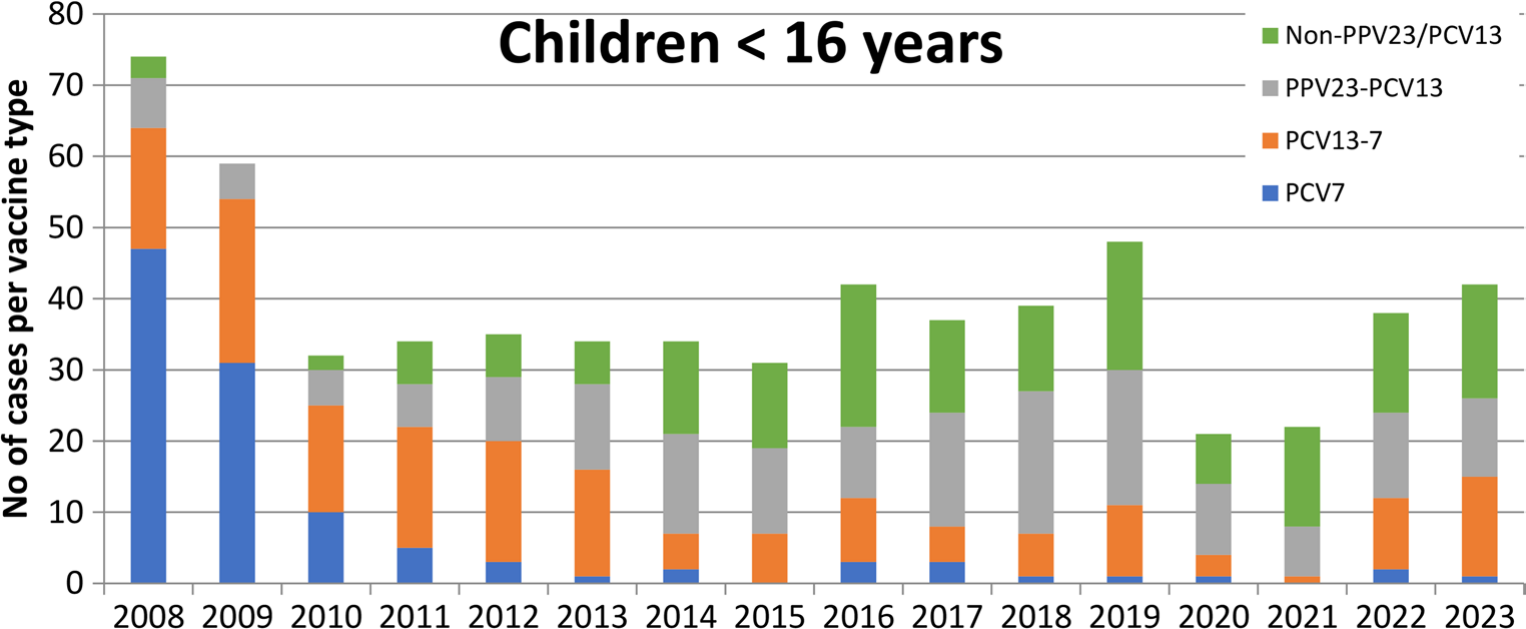
Annual cases of IPD by serotype in children < 16 in Ireland, 2008–2023 (data source: IMSRL)

While the numbers of IPD have decreased since the PCV introduction, there has been a growing trend towards non vaccine types (NVT) causing IPD. This was well documented by Miller et al.. across England and Wales during the replacement of the PCV7 with PCV13 [6]. More recent data confirm that, in the UK, NVT are the most common cause of IPD in children [7], with similar trends seen in Ireland [8] Detailed Irish data for the period from 2008 to 2023 is shown in Fig. 1 and reveals the same pattern of serotype replacement. Of note in 2021 21 of 22 cases of IPD in those < 16 years old was due to a non-PCV13 serotype. This suggests that clinicians should remain vigilant for IPD even in vaccinated children. In more recent years post pandemic the number of PCV7 and, in particular, some PCV13 only serotypes (PCV13-7 i.e. those not covered in PCV7), such as serotype 3 and 19 A have reemerged in children. With increased IPD in children, Immunisation Programs should ensure that uptake of the current PCV13 vaccine is not only maintained but returns to pre-covid-19 pandemic levels, as well as considering access to expanded vaccine serotypes. Specifically, our cases demonstrate serotypes outside of the two extended PCV15 and PCV20 vaccines, with only the serotype 20 covered in the polysaccharide vaccine (PPV23) that is recommended for older adults, and adults and children at increased risk of IPD. Looking at advances, PCV21, licensed for adults in the USA, is both under review for use in the EMA and under trial for use in high-risk children, includes the 20a, 23b, 31 serotypes - all present in our cases [9]. This again sparks debate on the decreased immunogenicity present with extended vaccine valency or “immunity creep”. A phenomenon whereby in introducing multiple serotypes there exists a reduction in an elicited immune response against any unique serotype.

Inborn errors of immunity are a well-recognised risk factor for IE and IPD diseases [3, 10]. It is standard practice at our institution to undertake a blood film (to screen for Howell-Jolly bodies) lymphocyte subsets, serum immunoglobulins, complement function assays for both classical and alternate pathways, ultrasound examination for the presence of a spleen and vaccine-specific antibody titres in all children who present with IPD. In certain cases, e.g. case 1, additional investigations including cytokine TLR/IL-1 studies; anti-cytokine antibodies and whole exome sequencing will be done in collaboration with the Immunology team. Case 2 had an isolated deficiency of the alternate complement pathway; however, they had adequate response to PCV13 vaccine serotypes indicating they would have been protected by a vaccine with activity against serotype 31.

## Conclusion

Paediatric IE is a rare but life-threatening condition which is very rarely attributable to *streptococcus pneumoniae.* The two cases which occurred at our institution within 12 months highlight the need for a high index of suspicion for IPD even in fully vaccinated children, the importance of access to expanded pneumococcal vaccination programs, and the role of host immunity in IPD.

## Supplementary Information

Below is the link to the electronic supplementary material.

Supplementary Material 1

Supplementary Material 2
