# Supplementary material for: Pneumococcal Infective Endocarditis in two vaccinated Irish children; The Challenge of Non-Vaccine Serotypes and the Role of Host Immunity
Source: Ir J Med Sci. 2026 Apr 9;195(3):1471–4. doi: 10.1007/s11845-026-04314-1 (PMC13341961; doi:10.1007/s11845-026-04314-1)
Supplement: Supplementary file 2 — Supplementary Material 2 [file 11845_2026_4314_MOESM2_ESM.pdf]

Authorship Form

Irish Journal of Medical Science

Springer

Manuscript ID Number: Not available

Article Title: (first few words) Pneumococcal Infective Endocarditis in two vaccinated Irish children; a host-pathogen equation

First Author: Bailey Crowley

E-mail: baileycrowley@gmail.com

AUTHORSHIP

I, the undersigned author(s), certify that:

- **I have seen and approved the final version of the manuscript, and all subsequent versions;**
- **I have made substantial contributions to conception and design, or acquisition of data, or analysis and interpretation of data;**
- **I have drafted the article or revised it critically for important intellectual content.**
- **I agree to be accountable for all aspects of the work in ensuring that questions related to the accuracy or integrity of any part of the work are appropriately investigated and resolved.**

I accept public responsibility for it, and believe it represents valid work. As an author of this article, I certify that none of the material in the manuscript has been previously published, nor is included in any other manuscript. I certify that this manuscript is not under consideration for publication elsewhere, nor has it been submitted or accepted in another publication in any form. The rights or interest in the manuscript have not been assigned to any third party.

Moreover, should the editor of *Irish Journal of Medical Science* request the data upon which the manuscript is based, I shall produce it. I also certify that I have read and complied with the copyright information, as found on the journal home page website.

After submission of this agreement signed by all authors, changes of authorship or in the order of the authors listed will not be accepted by Springer.

[x] Bailey Crowley
Author's signature

Bailey Crowley - 3/8/25
Printed name & date

☐
Author's signature

Printed name & date

☐
Author's signature

Bryony Treston - 3/8/25
Printed name & date

☐
Author's signature

Printed name & date

☐ S. Geoghegan
Author's signature

Sarah Geoghegan - 3/8/25
Printed name & date

☐
Author's signature

Printed name & date

☐
Author's signature

Printed name & date

☐
Author's signature

Printed name & date

Completed forms can be scanned and included as a pdf file during the online submission process as a supplemental file not for review, or submitted by fax to the editorial office: +91 44 42197763

Authorship Form

Irish Journal of Medical Science

Springer

Manuscript ID Number: Not available

Article Title: (first few words) Pneumococcal Infective Endocarditis in two vaccinated Irish children; a host-pathogen equation

First Author: Bailey Crowley

E-mail: baileycrowley@gmail.com

AUTHORSHIP

I, the undersigned author(s), certify that:

- **I have seen and approved the final version of the manuscript, and all subsequent versions;**
- **I have made substantial contributions to conception and design, or acquisition of data, or analysis and interpretation of data;**
- **I have drafted the article or revised it critically for important intellectual content.**
- **I agree to be accountable for all aspects of the work in ensuring that questions related to the accuracy or integrity of any part of the work are appropriately investigated and resolved.**

I accept public responsibility for it, and believe it represents valid work. As an author of this article, I certify that none of the material in the manuscript has been previously published, nor is included in any other manuscript. I certify that this manuscript is not under consideration for publication elsewhere, nor has it been submitted or accepted in another publication in any form. The rights or interest in the manuscript have not been assigned to any third party.

Moreover, should the editor of *Irish Journal of Medical Science* request the data upon which the manuscript is based, I shall produce it. I also certify that I have read and complied with the copyright information, as found on the journal home page website.

After submission of this agreement signed by all authors, changes of authorship or in the order of the authors listed will not be accepted by Springer.

☒ Bailey Crowley
Author's signature

Bailey Crowley
Printed name & date

☐
Author's signature

Printed name & date

☒ Ronan Leahy
Author's signature

Ronan Leahy
Printed name & date

☐
Author's signature

Printed name & date

☐
Author's signature

Printed name & date

☐
Author's signature

Printed name & date

☐
Author's signature

Printed name & date

☐
Author's signature

Printed name & date

Completed forms can be scanned and included as a pdf file during the online submission process as a supplemental file not for review, or submitted by fax to the editorial office: +91 44 42197763

Authorship Form

Irish Journal of Medical Science Springer

Manuscript ID Number: Not available

Article Title: (first few words) Pneumococcal Infective Endocarditis in two vaccinated Irish children: a host-pathogen equation

First Author: Bailey Crowley

E-mail: baileycrowley@gmail.com

AUTHORSHIP

I, the undersigned author(s), certify that:

- **I have seen and approved the final version of the manuscript, and all subsequent versions;**
- **I have made substantial contributions to conception and design, or acquisition of data, or analysis and interpretation of data;**
- **I have drafted the article or revised it critically for important intellectual content.**
- **I agree to be accountable for all aspects of the work in ensuring that questions related to the accuracy or integrity of any part of the work are appropriately investigated and resolved.**

I accept public responsibility for it, and believe it represents valid work. As an author of this article, I certify that none of the material in the manuscript has been previously published, nor is included in any other manuscript. I certify that this manuscript is not under consideration for publication elsewhere, nor has it been submitted or accepted in another publication in any form. The rights or interest in the manuscript have not been assigned to any third party.

Moreover, should the editor of *Irish Journal of Medical Science* request the data upon which the manuscript is based, I shall produce it. I also certify that I have read and complied with the copyright information, as found on the journal home page website.

After submission of this agreement signed by all authors, changes of authorship or in the order of the authors listed will not be accepted by Springer.

☒ Bailey Crowley
Author's signature

Bailey Crowley
Printed name & date

☐
Author's signature

Printed name & date

☐
Author's signature

AISLING FLINN 4/7/25
Printed name & date

☐
Author's signature

Printed name & date

☐
Author's signature

Printed name & date

☐
Author's signature

Printed name & date

☐
Author's signature

Printed name & date

☐
Author's signature

Printed name & date

Completed forms can be scanned and included as a pdf file during the online submission process as a supplemental file not for review, or submitted by fax to the editorial office: +91 44 42197763

Authorship Form

Irish Journal of Medical Science

Springer

Manuscript ID Number: Not available

Article Title: (first few words) Pneumococcal Infective Endocarditis in two vaccinated Irish children; a host-pathogen equation

First Author: Bailey Crowley

E-mail: baileycrowley@gmail.com

AUTHORSHIP

I, the undersigned author(s), certify that:

- **I have seen and approved the final version of the manuscript, and all subsequent versions;**
- **I have made substantial contributions to conception and design, or acquisition of data, or analysis and interpretation of data;**
- **I have drafted the article or revised it critically for important intellectual content.**
- **I agree to be accountable for all aspects of the work in ensuring that questions related to the accuracy or integrity of any part of the work are appropriately investigated and resolved.**

I accept public responsibility for it, and believe it represents valid work. As an author of this article, I certify that none of the material in the manuscript has been previously published, nor is included in any other manuscript. I certify that this manuscript is not under consideration for publication elsewhere, nor has it been submitted or accepted in another publication in any form. The rights or interest in the manuscript have not been assigned to any third party.

Moreover, should the editor of *Irish Journal of Medical Science* request the data upon which the manuscript is based, I shall produce it. I also certify that I have read and complied with the copyright information, as found on the journal home page website.

After submission of this agreement signed by all authors, changes of authorship or in the order of the authors listed will not be accepted by Springer.

☒ Bailey Crowley (signature)
Author's signature

Bailey Crowley - 3/8/25
Printed name & date

☐ [signature]
Author's signature

Dr Cilian Ó Maoldomhnaigh 28/07/2025
Printed name & date

☐
Author's signature

Printed name & date

☐ Patrick J Gavin, MD (signature)
Author's signature

Dr Patrick Gavin. 28/07/2025
Printed name & date

☐
Author's signature

Printed name & date

☐
Author's signature

Printed name & date

☐
Author's signature

Printed name & date

☐
Author's signature

Printed name & date

Completed forms can be scanned and included as a pdf file during the online submission process as a supplemental file not for review, or submitted by fax to the editorial office: +91 44 42197763

Authorship Form

Irish Journal of Medical Science Springer

Manuscript ID Number: Not available

Article Title: (first few words) Pneumococcal Infective Endocarditis in two vaccinated Irish children; a host-pathogen equation

First Author: Bailey Crowley

E-mail: baileycrowley@gmail.com

AUTHORSHIP

I, the undersigned author(s), certify that:

- **I have seen and approved the final version of the manuscript, and all subsequent versions;**
- **I have made substantial contributions to conception and design, or acquisition of data, or analysis and interpretation of data;**
- **I have drafted the article or revised it critically for important intellectual content.**
- **I agree to be accountable for all aspects of the work in ensuring that questions related to the accuracy or integrity of any part of the work are appropriately investigated and resolved.**

I accept public responsibility for it, and believe it represents valid work. As an author of this article, I certify that none of the material in the manuscript has been previously published, nor is included in any other manuscript. I certify that this manuscript is not under consideration for publication elsewhere, nor has it been submitted or accepted in another publication in any form. The rights or interest in the manuscript have not been assigned to any third party.

Moreover, should the editor of *Irish Journal of Medical Science* request the data upon which the manuscript is based, I shall produce it. I also certify that I have read and complied with the copyright information, as found on the journal home page website.

After submission of this agreement signed by all authors, changes of authorship or in the order of the authors listed will not be accepted by Springer.

☒ Bailey Crowley

Author's signature

Bailey Crowley

Printed name & date

☐ BFreyne

Author's signature

Bridget Freyne 29/07/2025

Printed name & date

☒ [signature]

Author's signature

ROBERT CUNNEY 31/07/2025

Printed name & date

☐

Author's signature

Printed name & date

☐

Author's signature

Printed name & date

☐

Author's signature

Printed name & date

☐

Author's signature

Printed name & date

☐

Author's signature

Printed name & date

Completed forms can be scanned and included as a pdf file during the online submission process as a supplemental file not for review, or submitted by fax to the editorial office: +91 44 42197763
